# Swallow Motor Pattern Is Modulated by Fixed or Stochastic Alterations in Afferent Feedback

**DOI:** 10.3389/fnhum.2020.00112

**Published:** 2020-04-09

**Authors:** Suzanne N. King, Tabitha Y. Shen, M. Nicholas Musselwhite, Alyssa Huff, Mitchell D. Reed, Ivan Poliacek, Dena R. Howland, Warren Dixon, Kendall F. Morris, Donald C. Bolser, Kimberly E. Iceman, Teresa Pitts

**Affiliations:** ^1^Department of Otolaryngology-Head and Neck Surgery, University of Louisville, Louisville, KY, United States; ^2^Kentucky Spinal Cord Injury Research Center, University of Louisville, Louisville, KY, United States; ^3^Department of Physiological Sciences, College of Veterinary Medicine, University of Florida, Gainesville, FL, United States; ^4^Department of Neurological Surgery, School of Medicine, University of Louisville, Louisville, KY, United States; ^5^Department of Medical Biophysics, Jessenius Faculty of Medicine, Comenius University, Bratislava, Slovakia; ^6^Robley Rex VA Medical Center, Louisville, KY, United States; ^7^Department of Mechanical and Aerospace Engineering, Herbert Wertheim College of Engineering, University of Florida, Gainesville, FL, United States; ^8^Department of Molecular Pharmacology and Physiology, Morsani College of Medicine, University of South Florida, Tampa, FL, United States

**Keywords:** deglutition, schluckatmung, diaphragm, facilitation, electrical stimulation, swallow, stochastic

## Abstract

Afferent feedback can appreciably alter the pharyngeal phase of swallow. In order to measure the stability of the swallow motor pattern during several types of alterations in afferent feedback, we assessed swallow during a conventional water challenge in four anesthetized cats, and compared that to swallows induced by fixed (20 Hz) and stochastic (1-20Hz) electrical stimulation applied to the superior laryngeal nerve. The swallow motor patterns were evaluated by electromyographic activity (EMG) of eight muscles, based on their functional significance: laryngeal elevators (mylohyoid, geniohyoid, and thyrohyoid); laryngeal adductor (thyroarytenoid); inferior pharyngeal constrictor (thyropharyngeus); upper esophageal sphincter (cricopharyngeus); and inspiratory activity (parasternal and costal diaphragm). Both the fixed and stochastic electrical stimulation paradigms increased activity of the laryngeal elevators, produced short-term facilitation evidenced by increasing swallow durations over the stimulus period, and conversely inhibited swallow-related diaphragm activity. Both the fixed and stochastic stimulus conditions also increased specific EMG amplitudes, which never occurred with the water challenges. Stochastic stimulation increased swallow excitability, as measured by an increase in the number of swallows produced. Consistent with our previous results, changes in the swallow motor pattern for pairs of muscles were only sometimes correlated with each other. We conclude that alterations in afferent feedback produced particular variations of the swallow motor pattern. We hypothesize that specific SLN feedback might modulate the swallow central pattern generator during aberrant feeding conditions (food/liquid entering the airway), which may protect the airway and serve as potentially important clinical diagnostic indicators.

## Introduction

During ingestion, the pharyngeal phase of swallow is initiated and regulated by a host of sensory afferents in the oral, pharyngeal, and laryngeal cavities (Pommerenke, 1928; Kahrilas and Logemann, 1993; Ertekin et al., 2000; Hiss et al., 2001; Humbert et al., 2009; Pitts et al., 2013; Spearman et al., 2014; Huff et al., 2018). Changes in temperature, food size and texture, or taste can significantly modulate the swallow motor pattern, and are used by speech-language pathologists as therapeutic options to treat swallowing disorders (dysphagia) (Bushmann et al., 1989; DePippo et al., 1992; Hiss et al., 2001; Kendall, 2002; Butler et al., 2004; Daniels et al., 2004, 2007; Clave et al., 2008; Troche et al., 2008; Humbert et al., 2009; Yamamura et al., 2010). While the clinical literature demonstrating these effects is robust, very little is understood about their mechanism of action. If these mechanisms are elucidated, specific sensory treatment parameters could be optimized for maximal therapeutic effect.

Historically, much of the basic investigation into the swallow pattern generator has been performed under *fictive* conditions (deafferented and paralyzed) using fixed-frequency electrical stimulation of the superior laryngeal nerve (SLN) (Gia, 1958; Doty, 1968; Miller, 1982; Dick et al., 1993). The SLN branches off the vagus nerve and provides both sensory and motor innervation to the larynx. While its stimulation can readily evoke a series of rhythmic swallows, it also strongly suppresses breathing, and there is limited information about how that pattern compares to a more natural stimulus condition. The key features of the swallow in an experimentally reduced preparation are a burst on the hypoglossal nerve followed by a burst on the vagus nerve (Gestreau et al., 1996, 2000; Roda et al., 2002; Bautista and Dutschmann, 2014; Bautista et al., 2014; Hashimoto et al., 2019). However, natural deglutition is complex and involves variable motor sequences produced by an array of muscles, and also includes inspiratory (e.g., diaphragm) muscle activation (“schluckatmung”) that is thought to produce negative intra-thoracic pressure to aid in propelling the bolus through the esophagus (Pitts et al., 2013, 2015a,b; Spearman et al., 2014). During normal breathing and swallow, laryngeal afferents are stimulated, producing variable sensory frequency patterns with discharge rates from 10 – 184 Hz, which are transmitted by the SLN (Storey, 1968; Bradley et al., 1983). This corresponds to stochastic-like afferent nerve firing discharge which can stimulate multiple behavior responses including apnea, swallow, and cough. Recent work has also demonstrated short-term facilitation of swallow duration (Horton et al., 2018) in response to SLN stimulation, which contrasts with the classical view of swallow as a strictly stereotypical motor event (Miller and Scheeington, 1916; Doty and Bosma, 1956; Doty, 1968; Miller, 1982). Additionally, the SLN carries afferent fibers which, which stimulated, can ultimately evoke laryngeal closure during swallow (Jafari et al., 2003), however this activity is not essential for the onset of the normal swallow sequence (Kitagawa et al., 2002). The vallecular space near the epiglottis is also innervated by SLN afferents (except in humans), and when food/liquid accumulates in this space behind the tongue, reflexive swallow occurs. When swallow is induced by delivering milk to the vallecular space of decerebrate piglets (Thexton et al., 2007, 2009), the motor pattern is modified by the presence of other rhythmic oral movements (suckling). This indicates that afferent feedback from natural stimuli can influence pharyngeal swallow motor pattern, and that this is brainstem-mediated.

Previous work has defined characteristics of swallow motor pattern based on deterministic repetitive SLN stimulation, and suggests that repetitive swallows are produced by changes in excitability of the swallow central pattern generator (CPG) (Jean, 2001). Previous studies have not systematically compared swallows evoked by electrical SLN stimulation to those evoked by a natural stimulus (e.g., water in the oropharynx or vallecula). Thus, it remains uncertain if the repetitive swallow motor patterns produced in response to SLN stimulation are directly comparable to a natural stimulus, or if the addition of variability in the stimulation parameters can be used experimentally to produce motor patterns akin to a natural stimulus. We tested the hypothesis that swallow-related upper airway and inspiratory (diaphragm and parasternal: schluckatmung) muscle activity is modified by use of fixed frequency and stochastic SLN electrical stimulation versus oral water infusion.

## Materials and Methods

Experiments were performed on four spontaneously breathing adult cats. Ethical approval of the protocol was confirmed by the University of Florida and University of Louisville Institutional Animal Care and Use Committees (IACUCs). The animals were initially anesthetized with sevoflurane (3–5%) via inhalation and then transitioned to sodium pentobarbital (35–40 mg/kg i.v.); supplementary doses were administered as needed (1-3 mg/kg i.v.). A dose of atropine sulfate (0.1–0.2 mg/kg, i.v.) was given at the beginning of the experiment to reduce airway secretions. Cannulas were placed in the femoral artery, femoral vein, and trachea. An esophageal balloon was placed via an oral approach to measure pressure in the mid-thoracic esophagus. Arterial blood pressure and end-tidal CO_2_ were continuously monitored. Body temperature was monitored and maintained at 37.5 ± 0.5 °C using a pad. Arterial blood samples were periodically removed for blood gas analysis. PO_2_ was maintained using air mixtures with enriched oxygen (25-60%) to maintain values above 100 mm Hg if needed.

Muscle activity was recorded via electromyography (EMG) using bipolar insulated fine wire electrodes according to the technique of Basmajian and Stecko (1962). Eight muscles were used to evaluate swallow occurrence: mylohyoid, geniohyoid, thyrohyoid, thyropharyngeus, thyroarytenoid, cricopharyngeus, parasternal, and costal diaphragm. These muscles span the actions during the pharyngeal phase of swallow: (a) mylohyoid, geniohyoid and thyrohyoid for hyolaryngeal elevation; (b) thyropharyngeus for inferior pharyngeal constriction; (c) cricopharyngeus for upper esophageal sphincter regulation; (d) thyroarytenoid for laryngeal adduction; and (e) parasternal and costal diaphragm for inspiratory (schluckatmung) activity (Pitts et al., 2013, 2015a,b; Spearman et al., 2014). As in our previous publications, swallow duration was defined as onset of the mylohyoid burst to the end of the thyropharyngeus burst.

Surgical placement of EMGs proceeded as follows: the digastric muscles were blunt dissected away from the surface of the mylohyoid and electrodes were placed medially in the left mylohyoid. A small horizontal incision was made at the rostral end of the right mylohyoid followed by an incision down the midline for approximately 5 mm to reveal the geniohyoid muscle. Electrodes were placed 1 cm from the caudal insertion of the geniohyoid muscle. The thyroarytenoid muscle electrodes were inserted through the cricothyroid window into the anterior portion of the vocal folds, which were visually inspected post-mortem. Minor rotation of the larynx and pharynx counterclockwise revealed the superior laryngeal nerve, which facilitated placement of the thyropharyngeus muscle electrodes. The thyropharyngeus is a fan shaped muscle with the smallest portion attached to the thyroid cartilage; electrodes were placed in the ventral, caudal portion of the muscle overlaying thyroid cartilage within 5 mm of the rostral insertion of the muscle. To place electrodes within the cricopharyngeus muscle, the larynx and pharynx were rotated counterclockwise to reveal the posterior aspect of the larynx. The edge of the cricoid cartilage was located by palpation and electrodes were placed in the cricopharyngeus muscle just cranial to the edge of this structure. Thyrohyoid muscle electrodes were inserted approximately 5 mm rostral to the attachment to the thyroid cartilage; those for the parasternal muscle were placed in the third intercostal space, just adjacent to the sternum, and the costal diaphragm EMGs were placed transcutaneously just under the xiphoid process. The positions of all electrodes were confirmed by visual inspection (following electrode placement and post-mortem) and by EMG activity patterns during breathing and swallow, as we have previously published (Pitts et al., 2013, 2015b, 2018; Spearman et al., 2014).

The right SLN was unilaterally exposed and bipolar hook electrodes were placed on the intact nerve. Voltage thresholds for evoking swallow were determined at the beginning of the experiment using fixed frequency (20 Hz) stimulus, and for the experimental condition the voltage was set at 1.5 times higher than threshold necessary for producing at least one swallow (4.4 ± 0.7 V). Non-deterministic stimulation frequencies were produced by a custom MATLAB (MathWorks; Natick, MA) program that shuffled inter-pulse intervals instantaneously corresponding to 4-40 Hz. Pulse parameters were controlled from a host PC interfaced to a custom electrical stimulator through a commercial interface board (QPID terminal board, Quanser; Markham, ON, Canada).

### Conditions

**FIGURE 1:**
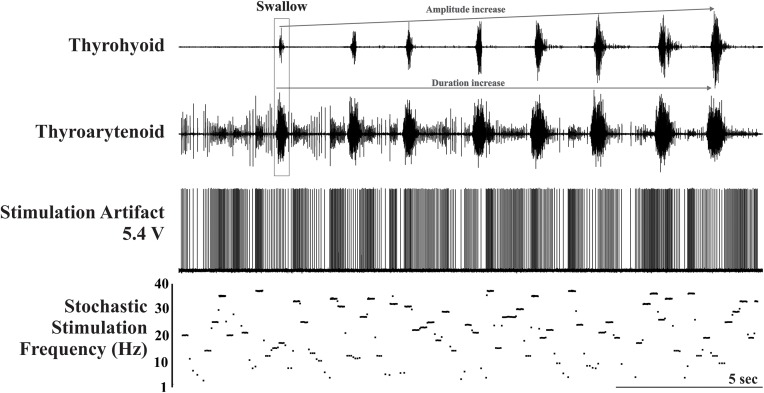
Example trial of the stochastic electrical stimulation condition illustrating the stimulation artifact and plot of stimulation frequency in Hz. Activity on the thyrohyoid marks the occurrence of the eight swallows. Note the amplitude and duration facilitation on the thyrohyoid EMG and the duration facilitation on the thyroarytenoid EMG. The stimulus artifact is also present on the thyroarytenoid EMG.

To initiate swallow via water, a bolus of approximately 3 ml was infused into the pharynx via a 1-inch long piece of polyethylene tubing (P.E. 90) (placed rostral to the faucial pillars) attached to 5 ml syringe. All water trials for each animal were performed by the same researcher to maintain stimulus consistency. Fixed frequency electrical stimulation was produced at 20 Hz, while the stochastic condition was produced across the range of 1-40 Hz (median of 20 Hz), each for 20 second series (see Figure 1). All stimuli were presented three times, separated by a minimum inter-stimulus interval of one minute; the presentation was randomized within each animal and across animals.

### Signal Analysis

Raw EMG signals were filtered (200-5000 Hz), rectified, and integrated with time constant of 20 ms. Swallow was identified by sequential bursts of the mylohyoid, geniohyoid, thyrohyoid, thyroarytenoid, and thyropharyngeus as well as a decline in tonic (followed by a burst) EMG activity of the cricopharyngeus (UES). Swallows that could not be differentiated from other behaviors (i.e., licking, cough, laryngeal elevation, laryngeal adductor reflex and aspiration reflex) were excluded from analysis. To avoid analyzing data from spontaneous swallow activity, the EMG activity was included in the analysis if the swallow occurred within 30 seconds of the initial water or within the stimulus duration. Reported maximum EMG values were calculated as a percentage of maximum for each muscle across the experiment for normalization across animals (i.e., the maximum EMG amplitude for each muscle was 100%).

### Statistical Analysis

A mean ± standard deviation (SD) was calculated for each measure and animal including all induced swallows, and then averaged for each condition across animals (Table 1). For statistical analysis of group differences by *condition*, an ANOVA with Fisher’s least significant difference *post hoc* tests were performed as appropriate (Table 1). To assess *short-term facilitation*, a repeated-measures ANOVA was performed comparing the first swallow in the series to each subsequent swallow with Fisher’s least significant difference *post hoc* tests performed as appropriate, similar to procedures performed by Horton and colleagues (2018) (Table 1). A difference was considered significant if the *p*-value was less or equal to 0.05. To assess relationships between changes in EMG amplitude and duration during swallow, Pearson’s product moment correlations (*r*) were calculated comparing all amplitude measures across conditions (Table 2).

**TABLE 1:** Means and standard deviations (SD) for EMG amplitude (% maximum) and total swallow duration changes across the three conditions (ANOVA) and evidence of short-term facilitation (repeated-measures ANOVA).

		Mylolyoid	Geniohyoid	Thyrohyoid	Thyroarytenoid	Thyropharyngeus	UES (Cricopharyngeus)	Parasternal	Costal diaphragm	Swallow duration
		Mean ± SD	Mean ± SD	Mean ± SD	Mean ± SD	Mean ± SD	Mean ± SD	Mean ± SD	Mean ± SD	Mean ± SD
**Water (W)**	**SW#**									
	1	63 ± 16	49 ± 33	59 ± 20	66 ± 12	44 ± 38	63 ± 28	77 ± 32	75 ± 23	588 ± 121
	2	69 ± 15	34 ± 23	57 ± 38	86 ± 19	49 ± 35	50 ± 16	70 ± 41	76 ± 28	561 ± 84
	3	55 ± 8	26 ± 19	42 ± 23	66 ± 12	54 ± 46	78 ± 20	50 ± 12	87 ± 15	423 ± 87
		**62 ± 13**	**36 ± 25**	**53 ± 27**	**73 ± 14**	**49 ± 39**	**64 ± 22**	**66 ± 28**	**79 ± 22**	**524 ± 98**
**20 Hz fixed (F)**										
	1	53 ± 10	72 ± 20	57 ± 17	71 ± 8	34 ± 28	37 ± 25	36 ± 23	20 ± 21	605 ± 81
	2	78 ± 10*	68 ± 7	68 ± 7	63 ± 6	49 ± 37	39 ± 22	29 ± 13	22 ± 22	612 ± 95
	3	76 ± 10*	75 ± 18	76 ± 6	64 ± 3	46 ± 31	46 ± 22	42 ± 23	20 ± 22	726 ± 122*
	4	82 ± 13*	74 ± 11	80 ± 10	73 ± 13	57 ± 37	51 ± 32	40 ± 23	20 ± 20	731 ± 95*
	5	76 ± 7*	82 ± 14	81 ± 15	66 ± 10	57 ± 29	49 ± 21	50 ± 35	19 ± 20	830 ± 120*
		**73 ± 10**	**74 ± 14**	**73 ± 11**	**67 ± 8**	**49 ± 32**	**44 ± 24**	**39 ± 23**	**20 ± 21**	**701 ± 103**
**1–20 Hz variable (V)**										
	1	62 ± 17	72 ± 20	51 ± 4	63 ± 4	41 ± 29	42 ± 13	50 ± 31	21 ± 20	557 ± 29
	2	62 ± 13	67 ± 3	67 ± 3	69 ± 10	52 ± 22	46 ± 20	46 ± 30	21 ± 21	644 ± 69
	3	70 ± 17	76 ± 20	82 ± 17*	68 ± 10	50 ± 22	52 ± 20	53 ± 36	20 ± 21	644 ± 38*
	4	80 ± 14	78 ± 17	87 ± 13*	79 ± 20	69 ± 13*	53 ± 23	47 ± 33	19 ± 21	724 ± 116*
	5	82 ± 6	76 ± 13	77 ± 15*	72 ± 9	82 ± 16*	52 ± 31	49 ± 29	19 ± 20	794 ± 160*
	6	76 ± 9	77 ± 15	78 ± 10*	75 ± 6	73 ± 5*	57 ± 34	46 ± 36	19 ± 20	785 ± 90*
		**74 ± 14**	**72 ± 13**	**73 ± 10**	**70 ± 10**	**68 ± 16**	**57 ± 23**	**51 ± 31**	**21 ± 21**	**691 ± 84**
p value**		**0.01**	**<0.001**	**0.001**	0.8	**0.03**	0.2	**0.01**	**<0.001**	0.08
*Post hoc*	W vs. F	**0.003**	**<0.001**	**0.001**		0.6		**0.003**	**<0.001**	
	W vs. V	**0.008**	**<0.001**	**0.001**		**0.03**		0.2	**<0.001**	
	F vs. V	0.6	0.6	0.9		0.2		0.3	0.9	

Swallow number (SW#) is expressed as 1 to *n*; condition means and SD are represented in bold below each condition; ANOVA *p*-values and significant *post hoc* tests are expressed at the bottom; significant short-term facilitation is expressed as a * to the right of the swallow measure. *Facilitation analysis: significant change compared to the first swallow in the series, **Significant change by condition. Significant if *p* < 0.05; significant values are bolded.

**TABLE 2:** Pearson correlations comparing EMG amplitudes (% maximum) and the total swallow duration (ms).

	MyHy	GeHy	ThHy	ThAr	ThPh	UES	PS	Cos Dia	Duration
MyHy		**0.48**	**0.48**	0.13	0.27	0.01	–0.17	–0.22	0.33
GeHy			0.39	0.09	–0.06	0.01	–0.24	–0.39	**0.57**
ThHy				0.27	0.12	–0.11	–0.32	–0.38	0.29
ThAr					–0.15	–0.16	–0.17	–0.1	0.15
ThPh						–0.04	–0.2	–0.33	0.01
UES							**0.54**	**0.49**	0.09
PS								**0.61**	–1.7
Cos Dia									0.22

*All data were pooled across the three conditions: water, fixed and stochastic electrical stimulation. Moderately strong relationships (in bold) are present among the upper airway muscles (geniohyoid and thyrohyoid with the mylohyoid) and among the inspiratory muscles (parasternal and costal diaphragm) with the upper esophageal sphincter. [MyHy = mylohyoid; GeHy = geniohyoid; ThHy = thyrohyoid; ThPh = thyropharyngeus; UES = upper esophageal sphincter (aka cricopharyngeus); PS = parasternal; Cos Dia = costal diaphragm; and Duration = total swallow duration in ms].*

## Results

All three stimulus conditions produced apnea and repetitive swallows: water (3.2 ± 0.5), fixed frequency (7.5 ± 2.6), and stochastic (9.2 ± 2.5) electrical stimulation. Of note, the stochastic stimulation produced significantly more swallows compared to the fixed frequency stimulation (*p* = 0.005), due to a difference in time from the initiation of the stimulation to the first swallow [fixed (4.2 ± 0.9 s), stochastic (2.7 ± 0.4 sec; *p* = 0.01)].

**FIGURE 2:**
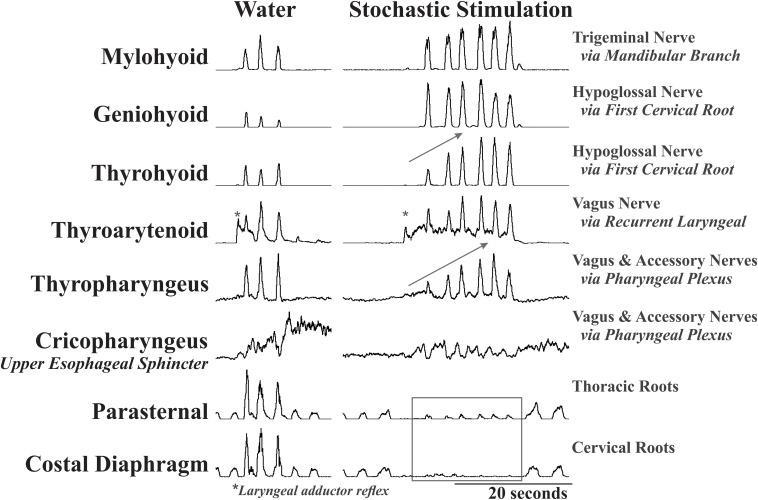
Representative EMG examples of repetitive swallow during water infusion and with stochastic electrical stimulation of the SLN. This example demonstrates the effect of condition on EMG amplitude with a global increase in mylohyoid, geniohyoid, thyrohyoid and a decrease in the parasternal and costal diaphragm. Additionally, the gray arrows indicate the short-term facilitation across the swallow series on the thyrohyoid and thyropharyngeus. Note the differential response of the geniohyoid and thyrohyoid to the stimulation even though both are innervated by the same nerve. *Labels a laryngeal adductor reflex. All EMGs in this figure have been integrated, and the tonic activity on the thyroarytenoid EMG is stimulus artifact.

**FIGURE 3:**
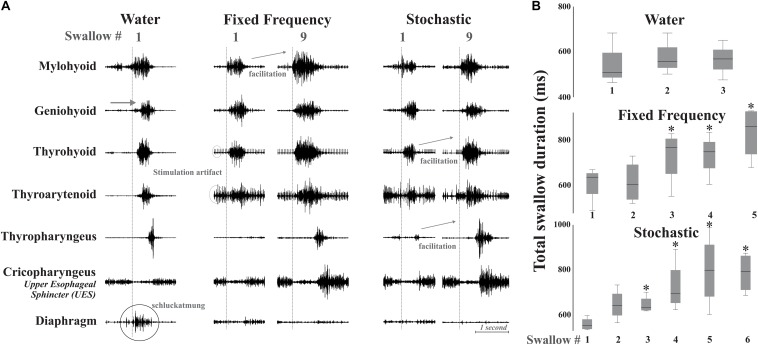
**(A)** Example of changes in the swallow pattern produced by fixed and stochastic frequency stimulation with a water swallow for comparison. There are small changes in EMG initiation with the stimulation conditions. The vertical dotted line marks onset of the relaxation of the upper esophageal sphincter (UES), which allows the bolus to pass into the esophagus. Diaphragm activity (termed “schluckatmung”) is present during the water swallow. Short-term amplitude facilitation from the first to the ninth swallow is seen in EMGs of the mylohyoid with fixed frequency stimulation, and in the thyrohyoid and thyropharyngeus with stochastic stimulation. Artifact is present on EMGs with stimulation. **(B)** Box plots of swallow durations for bouts of repeated swallows, during different simulation conditions (box heights are standardized across all three conditions). *Represents significance *p* < 0.05.

There was a significant effect of condition on the EMG amplitude (percent of maximum) of mylohyoid, geniohyoid, thyrohyoid, thyropharyngeus, parasternal, and costal diaphragm (Table 1). Electrical stimulation (fixed and stochastic) increased the mylohyoid (∼120%), geniohyoid (∼200%), and thyrohyoid (137%) amplitude compared to water (Figures 1–3). Stochastic stimulation also increased thyropharyngeus amplitude by 138% compared to water (Figures 1–3). Conversely, there was significant depression of the costal diaphragm EMG amplitude by electrical stimulation (fixed and stochastic; ∼75%) and of the parasternal by fixed electrical stimulation (41%) compared to water (Table 1 and Figures 2, 3). There was no significant effect of condition on swallow duration (Table 1 and Figure 3).

There was evidence of short-term facilitation with fixed electrical stimulation (i.e., significant increase in EMG amplitude when compared to the first swallow in the series; see Figure 3 and Table 1) on the mylohyoid (*p* = 0.01) with significant increases starting at the second swallow (*p* = 0.004), continuing through the third (*p* = 0.006), fourth (*p* = 0.001), and fifth (*p* = 0.007) in the series (Table 1). During stochastic stimulation there was evidence of short-term facilitation on the thyrohyoid (*p* = 0.02) starting at the third swallow (*p* = 0.006), continuing through the fourth (*p* = 0.002), fifth (*p* = 0.01) and sixth (*p* = 0.01); and the thyropharyngeus (*p* = 0.05) starting at the fourth swallow (*p* = 0.05), continuing through the fifth (*p* = 0.008) and sixth (*p* = 0.02) in the series (Table 1).

For swallow duration there was evidence of short-term facilitation with fixed and stochastic stimulation (Figure 3; Table 1). Significant increases in swallow duration with fixed stimulation (*p* = 0.005) started at the third swallow (*p* = 0.02), continuing through the fourth (*p* = 0.02) and fifth (*p* = 0.005); stochastic stimulation facilitation (*p* = 0.03) started at the third swallow (*p* = 0.001), continuing through the fourth (*p* = 0.04), fifth (*p* = 0.04) and sixth (*p* = 0.01) swallow in the series.

Table 2 is a matrix showing all Pearson Product moment correlations for EMG amplitudes and swallow duration combined across all conditions. This analysis resulted in six moderate correlations: mylohyoid and geniohyoid (*r* = 0.48); mylohyoid and thyrohyoid (*r* = 0.54); UES (cricopharyngeus) and parasternal (*r* = 0.49); UES (cricopharyngeus) and costal diaphragm (*r* = 0.54); parasternal and costal diaphragm (*r* = 0.61); and geniohyoid to swallow duration (*r* = 0.57).

## Discussion

Modulation of afferent feedback is an important component in determining the stability of a reflexive motor pattern. This is the first study to demonstrate the differential effects of water infusion vs. electrical stimulation (stochastic or fixed) of the SLN on swallow production. The effects of electrical stimulation included significant increases in upper airway muscle (mylohyoid, geniohyoid, thyrohyoid, and thyropharyngeus) EMG amplitudes, and significant depression of the schluckatmung activity evidenced by the decreases in diaphragm (fixed and stochastic) and parasternal (fixed) EMG amplitudes. Additionally, there is evidence of short-term amplitude facilitation of the mylohyoid with fixed frequency stimulation, and of the thyrohyoid and thyropharyngeus with stochastic frequency stimulation.

### Fixed Versus Stochastic Stimulation

Our observations suggest that the stochastic stimulation increased excitability in the swallow CPG as evidenced by a reduction in the time to the first swallow, increases in EMG amplitudes, and an increase in number of swallows produced. Beyak et al. (1997) demonstrated that an increase in fixed stimulation frequency shortened latency and increased swallow number. Our data suggest that adding variance to the electrical stimulation signal works similarly, and maintains the overall stimulation delivered. The variance (i.e., noise) in an electrical signal has been shown to both increase information content of a signal and increase detection of a weak signal in sensory systems (Moss et al., 2004). Potential applications that have been explored could include stabilizing breathing (Paydarfar et al., 1986, 2006) and the suck-swallow patterns (Finan and Barlow, 1998) in pre-term infants, and in decreasing tremor and bradykinesia in parkinsonian patients and rats using deep brain stimulation (Grill et al., 2001; Brocker et al., 2017). Historically, studies in deglutition have used only fixed frequency stimulation between 5 and 30 Hz, with a range of 20–30 Hz optimally evoking rapid continuous swallow with short latencies and low threshold periods (Bieger et al., 1977; Kessler and Jean, 1985; Ezure et al., 1993; Oku et al., 1994; Ootani et al., 1995; Sang and Goyal, 2001; Kitagawa et al., 2009). In the current study, we chose the parameters to stimulate at a 20 Hz fixed frequency or with a stochastic pattern where the average was around 20 Hz using a custom electrical stimulator which appeared to add a sufficient amount of “noise” to the signal without degrading the sensory input.

In clinical dysphagia therapy, surface electrical stimulation (i.e., NMES) uses an 80 Hz fixed frequency (Poorjavad et al., 2014), and has been successful in short term sessions (Humbert et al., 2006; Ludlow et al., 2007), but has been unsuccessful for long term therapeutic uses (Carnaby-Mann and Crary, 2007; Shaw et al., 2007; Bülow et al., 2008; Bogaardt et al., 2009; Christiaanse et al., 2011). While the voltage and intensity of the stimulus have been scrutinized, the frequency parameters have not. We believe that the current study is the first of many that are needed to begin testing and optimizing alterations in stimulation frequency for effective swallow manipulation.

### Diaphragm Activity During Swallow

Our recent work has extended the hypothesis that the swallow pattern generator activates motoneuron pools in the spinal cord for contraction of diaphragm and parasternal muscles (Spearman et al., 2014; Pitts et al., 2015a, b). This component of the swallow pattern has been described by research groups since the 1800’s, however, there have been very few studies in the modern era. Rosenthal (1861) was the first to report on the diaphragm contractions during SLN stimulation, and Arloing (1874) was the first to provide substantial evidence that these contractions form an active part of the swallow pattern by creating a negative deflection. The field termed this activity schluckatmung: a German word meaning “swallow-breath”. While this may not be the optimal term to describe this activity, it has continued to be used in the ensuant literature, for example Marckwald (1888) and Bosma (1957), Forester and colleagues (Feroah et al., 2002a, b; Bonis et al., 2011). In recent years, inspiratory motor drive during swallow has been reported in humans (Wilson et al., 1981; Hardemark Cedborg et al., 2009) and animals (Gestreau et al., 1996; Bonis et al., 2011). Previous work by our group showed that parasternal activity could be modulated through varying swallow stimuli, and that mechanical stimulation of the oropharyngeal wall produced parasternal EMG contractions with increased amplitude and duration (Spearman et al., 2014).

Our hypothesis is that breathing and swallow both involve an aspiration pump: they use negative pressure to “suck” air/bolus into the thoracic cavity (swallow also has significant positive pressure from above the bolus). This is consistent with McConnel’s theory of the “hypopharyngeal suction pump” although we hypothesize this pressure is created by inspiratory muscle activation instead of laryngeal elevation (McConnel et al., 1987, 1988a,b,c,d; McConnel, 1988). Our current results demonstrating that electrical SLN stimulation depresses diaphragm activity during swallow reveal why this was previously regarded as a non-existent and/or un-remarkable portion of the swallow pattern, as it would not have been apparent to most investigators using those common experimental conditions. This effect also mirrors what has been observed in the field of respiratory control. Bellingham and colleagues (Bellingham et al., 1989) were the first to demonstrate that SLN stimulation induced central apnea via a chloride-dependent oligo-synaptic pathway which hyperpolarizes phrenic motoneurons. More recently, Pilowsky and colleagues (Sun et al., 2011) demonstrated further effects in the core respiratory CPG by activation of expiratory-decrementing neurons in the ventral respiratory group, which in turn inhibit initiation of inspiration. However, we have demonstrated that using water as a stimulus for swallow can result in swallow occurrence across all phases of respiration (Pitts et al., 2013, 2015b; Spearman et al., 2014). We also previously reported that, in a non-paralyzed freely breathing animal with water as the stimulus, 16 of 18 inspiratory cells in the NTS were active during swallow and 7 of these increased their firing frequency (Pitts et al., 2018). We theorize that, while SLN stimulation does produce repetitive swallow, it may also through a secondary mechanism produce apnea (or inhibition of inspiratory neuronal activity).

### Facilitation of Upper Airway Muscles

Short-term plasticity of excitatory synapses has been extensively studied in both invertebrates and vertebrates (Atwood and Karunanithi, 2002; Pan and Zucker, 2009). It is characterized by an enhancement of synaptic transmission after a prolonged period of stimulation, and can affect the function of neural circuitry. Potential mechanisms of short-term synaptic enhancement in swallow were elegantly explained in the recent paper by Horton et al. (2018). They propose that, because of the importance of the nucleus tractus solitarius (NTS) in the swallow motor pathway, several mechanisms could be involved, including glutamatergic axon terminals (Fortin et al., 1992), activation of N-methyl-D-aspartate receptors within the NTS (England et al., 1992), and neurotransmitters such as nicotine (Kalappa et al., 2011), serotonin, and/or glutamate (Bieger, 1981; Hashim and Bieger, 1987). The results from the current study also suggest that facilitation is present or modulated downstream from the NTS. Our selection of EMGs allows analysis of muscles that are innervated by the same cranial nerve(s). For example, geniohyoid and thyrohyoid are both innervated by the hypoglossal nerve via the first cervical root. While both had significant increases in EMG amplitude during SLN stimulation compared to water (geniohyoid by ∼200% and thyrohyoid by 137%), the thyrohyoid also demonstrated short-term facilitation throughout the swallow series during variable stimulation (Figure 1). Of all the upper airway muscles measured, geniohyoid showed the greatest change in amplitude with SLN stimulation. Interestingly, in the decerebrate pig studies, geniohyoid was the only muscle that showed consistent modulation during swallow in the presence vs absence of natural background rhythmic oral feeding movements (Thexton et al., 2007, 2009).

Along with Horton et al. (2018), our current results contradict the classic view that SLN stimulus-induced swallows have a strictly stereotypical motor action, and instead suggest that swallow is strongly shaped by frequency and location-dependent afferent information. Many modern studies also report significant variance in swallow amplitude. Doty and Bosma (1956), observed intra-muscle and inter-animal electromyography (EMG) variability with swallow evoked by mechanical or electrical stimulation in dogs, cats, and monkeys. Although not directly indicated, irregular EMG activity was also reported in certain muscles when attempts were made to alter the temporal pattern of swallow (e.g., fixing hyoid, applying lingual traction, opening mouth, etc.). Studies by Thexton et al. (2007, 2009) demonstrated that contractions of certain swallow muscles are prone to increased variability due to the diversity of fiber types and the multiple functions they serve throughout different phases of swallow, and that a “switch” between activation of fiber types/functions can be provoked by a change in stimulus condition. In light of these findings, interpretation of swallow data obtained under SLN electrical stimulation conditions may need to be re-examined.

While electrical SLN stimulation does trigger swallow, those swallows are highly modified compared to those of a naturally induced behavior. From a clinical perspective, stimulation of the larynx is aberrant, as it would occur if food or liquid had entered the airway. Thus, the depression of diaphragm activity and increase in the upper airway muscle activation that we observed during SLN stimulation may act as protective mechanisms that would reduce negative pressure on the bolus, increase pharyngeal clearance, and therefore decrease further aspiration risk. This is important for interpretation of studies on swallow using reduced animal preparations.

### Limitations of the Experimental Design

The greatest limitation of the experimental design was the use of anesthesia, and the consequent potential suppression of airway reflexes. This was chosen because, in anesthetized animals, continuous SLN stimulation inhibits breathing (Donnelly and Haddad, 1986). Our choice of experimental preparation allowed for a train of swallows to be produced without potential interference from the breathing central pattern generator. Although it is clear that conditional modulation of the swallow motor pattern is possible in decorticate animals (Thexton et al., 2007, 2009), the current results cannot be directly extrapolated to swallow function in awake animals, because anesthesia reduces cortical function. Additionally, we did not direct record from any muscles which are innervated by the hypoglossal motor nucleus, because the geniohyoid is innervated via a cervical root. While we do see pre-swallow oral behaviors in response to water infusion, we did not observe such behaviors with electrical SLN stimulation, and do not know how they would have influenced swallow under those conditions.

## Conclusion

Electrical stimulation of the SLN produces trains of swallows with evidence of short-term facilitation of specific EMG amplitudes and swallow duration. When compared to the fixed frequency stimulus, stochastic stimulation increased the excitability of the swallow pattern generator without changing overall current delivered. SLN stimulation significantly depressed diaphragm and parasternal (inspiratory muscles) activity during swallow, implicating involvement of spinal pathways.

## Data Availability Statement

The datasets generated for this study are available on request to the corresponding author.

## Ethics Statement

The animal study was reviewed and approved by the University of Louisville IACUC.

## Author Contributions

TS, MM, IP, WD, KM, DB, and TP conceptualized and designed the data. SK, TS, MM, AH, MR, IP, DH, KM, DB, KI, and TP acquired, analyzed, and/or interpretated the data. SK, TS, MM, AF, MR, IP, DH, WD, KM, DB, KI, and TP drafted, revised, and/or approved the manuscript.

## Conflict of Interest

The authors declare that the research was conducted in the absence of any commercial or financial relationships that could be construed as a potential conflict of interest.
